# Circadian clocks: It’s time for chronobiology

**DOI:** 10.1371/journal.pbio.3002426

**Published:** 2023-11-27

**Authors:** Martha Merrow

**Affiliations:** LMU Munich, Faculty of Medicine, Institute of Medical Psychology, Munich, Germany

## Abstract

Circadian clocks are everywhere, yet we still have not translated the vast knowledge on the properties of circadian clocks into practical applications. This Perspective looks at the past, present and future of chronobiology.

## Teaser

Circadian clocks are everywhere, yet we still have not translated the vast knowledge gained in the past 20 years on the properties of circadian clocks into practical applications. We are missing opportunities for improving quality of life, health, and sustainability.

This article is part of the *PLOS Biology* 20th Anniversary Collection.

In almost every paper I write and every talk I give, I emphasize, right at the beginning, the pervasiveness of the circadian clock. I do this as a challenge to the imagination: “pervasive” is vague enough to make most people wonder, “in what sense or dimension?” And the answer here is, “in almost any dimension you can imagine.” How can this be?

Circadian clocks are molecular machines that organize daily timing. They all share a set of properties shaped by evolution. Circadian clocks entrain or synchronize using highly predictable signals in the environment, called zeitgebers, which essentially tell what time it is. The most reliable zeitgeber is light and dark, but other signals such as temperature are also highly regular. Endogenous signals (for instance, circulating hormones) are also setting the clock at the cellular level. Given the right set of constant conditions, circadian clocks will show an approximately 24 hour free running period. This makes an easy demonstration of the endogenous nature of the clock.

So how is it that clocks are pervasive? They are pervasive in that they operate from the level of molecules to that of behavior, in possibly all cells within an organism, and in organisms from all kingdoms of life. They are well known in eukaryotes and in the photosynthetic prokaryotes called cyanobacteria [1]. Recently, they have also popped up in non-photosynthetic bacteria, which is possibly a last frontier in the field [2,3]. Thus, pervasive, as an adjective, works at almost any level you can imagine. This observation suggests that the circadian clock must be important, and we have hints regarding this from the consequences of behaviorally or genetically suppressing the clock (for instance, increased risk of cancer from shift work [4], increased body mass index from social jetlag [5], and metabolic disease in clock mutant animals [6]).

The past 2 decades have been rich with new information on the circadian clock on many levels. Molecular mechanisms have been clarified, and the complicated network of feedbacks that lead to a self-sustained circadian clock is being worked out. Most clock components have been identified using forward genetic screens. When components are identified via genetic means, they are discoverable and verifiable, but if they require nongenetic tools, identification becomes a much more difficult task, and there is evidence that such clock components exist [7]. We know that, despite the mouse being a nocturnal animal, the clock in humans parallels many observations in mice. We have learned in no uncertain terms that an intact clock is a fitness trait: break the clock at your own peril, as your neighbor will quickly overtake you [8,9]!

What is left to know about the circadian clock? Most things that you study will have some clock regulation somewhere, so the list is long. But for card-carrying chronobiologists, here are a few things to think about with regard to circadian organization, sleep and health, and climate change.

If we are a mere collection of oscillating cells, each entrained or synchronized by multiple zeitgebers, how is it that all of the individual parts are oscillating with an optimal phase relative to one another? We hypothesize that the illness that follows from long-term shift work, or even from short-term jetlag, stems from misalignment or lack of synchrony of all the circadian clocks. In my opinion, we do not know enough about what optimal phase alignment is, how the individual clocks respond in misalignment conditions, how in-tissue pacemaker functions are organized (which tissues drive synchrony of others), or how far we can stretch the system (what are the degrees of freedom?) vis a vis cellular entrainment. What evolved to accommodate photoperiodism—entraining differently according to a systematically changing light/dark cycle—might also determine the variety of internal phase relationships that are tolerated, and could thus facilitate, for instance, jetlag. The development of biomarkers [10] is a hot topic in our field. If they are tuned to address not only organismal phase but also tissue and cell-specific phases, they could be used to quantify misalignment and thus allow implementation of knowledge of circadian clocks into preventative health. Objective assessment of chronotype and degree of synchronization qualifies as a form of personalized medicine. There are already clinics and protocols that implement “circadian medicine,” but they address only the tip of the iceberg relative to what is possible. Furthermore, because of the focus on clock components discovered using genetic tools, we tend not to think in terms of clock structures [11], which will undoubtedly be part of the full description of circadian organization.

One of the most obvious clock-regulated behaviors in humans is the timing of sleep. In order to get into the human clock, the circadian clocks field has piggybacked on sleep research in a big way. Recent studies have used data collected in Biobanks—genotyping in combination with questions concerning sleep time or, even better, actigraphy data showing detailed, daily behavior in up to 100,000 individuals [12]—to discover correlations between the timing of behavior and genetic regulation of sleep and the circadian clock. Scientists in Japan are organizing a Sleep Check, which will be incorporated into the annual mandatory health checkup for the entire population (H. Ueda, personal communication). These sorts of projects have the potential for massive impact. Quality of life and health fail if sleep is poor. Conversely, poor sleep can be an indication of illness, possibly acting as an early warning system [13]. Collecting sleep parameters is an easy way to aid understanding of the clock and sleep and to figure out how to use sleep as a diagnostic tool. To that end, smart watches could be used to collect massive amounts of data that could extend our understanding of the many nuances of sleep and clock, such as how the timing, duration, and consolidation of sleep differs in young, middle aged, and older individuals.

Moving from the organismal to the systems level, it’s a painful reality that people on the Earth are starving. This stems in part can stem from challenges in agriculture at the local level, for example, due to arid or flooded conditions, poor soil, pathogens, and climate change. Crop productivity in the best of conditions is regulated in part by flowering time, which is mediated by the circadian clock, photoperiod, and seasonal temperature sensing [14]. Climate change is acting to impact crop production by changing seasonal temperature fluctuations and increasing the frequency of extreme weather events, both of which can modify flowering time. By targeting the plant circadian clock with a combination of conventional and precision breeding and enhancements to soil—in order to adjust to the flowering time and or to rewire clock regulated stomatal closure to reduce water loss—chronobiology can and should contribute to feeding the world.

Research in circadian biology is far from finished. Answers to some of the issues mentioned above will demonstrate just how hardwired the circadian clock is in basic molecular, cellular, organismal, and community structures. Unfortunately, chronobiology sometimes moves slowly: Our experiments are intensive and expensive, requiring prolonged sampling over hours, days, and even over seasons and years. But a complete understanding of biology will never be possible if we do not work at all of these timescales. For comparison, think of how much more information a movie delivers relative to a snapshot: It fills in the context and leads to a fuller understanding. This is what chronobiology can contribute to biology.
